# Osteoid Osteoma of the Trapezium: A Rare Case Report and Literature Review

**DOI:** 10.7759/cureus.48889

**Published:** 2023-11-16

**Authors:** Konstantinos Koutas, Spyridon Papagiannis, Vasileios Giannatos, Theodoros Stavropoulos, Zinon Kokkalis

**Affiliations:** 1 Orthopaedics and Traumatology, University General Hospital of Patras, Patras, GRC; 2 Orthopaedics and Traumatology, Patras University Hospital, Patra, GRC; 3 Orthopaedics, University General Hospital of Patras, Patras, GRC; 4 Orthopaedic Department, Universtity of Patras, Patras, GRC; 5 Orthopaedic Surgery, Medical School, University of Patras, Patras, GRC

**Keywords:** benign bone lesions, open excision, wrist pain, trapezium, osteoid osteoma

## Abstract

Osteoid osteoma is the most common benign osteogenic bone neoplasm. Osteoid osteomas are typically located in the metaphysis and diaphysis of long bones, especially the tibia and femur. However, less common sites of the skeleton can be affected as well, including carpal bones. Among carpal bones, the scaphoid and the capitate are the most affected. Osteoid osteoma of the trapezium is an extremely rare entity, with only seven cases reported in recent literature. We present a case of a 29-year-old male with persistent left wrist pain who was diagnosed with an osteoid osteoma of the trapezium bone. The diagnosis was based on the patient’s history, clinical examination and findings from the CT scan, MRI, and plain radiographs. The patient was treated with an excision biopsy with no additional bone grafting. After a follow-up period of 12 months, no pain or signs of recurrence were present. We conducted a literature review to elucidate the clinical presentation as well as the proper diagnostic tools and therapeutic methods for this rare occurrence.

## Introduction

Osteoid osteoma, which Jaffe first reported in 1935 [1], is a moderately frequent skeletal lesion that accounts for roughly 12% of all benign bone neoplasms. These tumors usually manifest in the cortises of long bones, such as the femur and the tibia, which are the most common sites of presentation [2-4]. Reviewing the literature, we found only seven osteoid osteomas reported at the trapezium. The patients typically complain of pain worsening during the night that may also be intense enough to awaken them [3,5,6]. A computed tomography (CT) scan is the imaging of choice to reach a diagnosis, considering plain radiographs may be inconclusive. Findings ought to describe a sclerotic nidus surrounded by a radiolucent halo. The definitive diagnosis is set by histological examination. Treatment is mainly surgical, since most of the osteoid osteomas of the carpus are easily accessible. Conservative treatment must be considered in patients where the loci of the lesions are difficult to approach [3].

## Case presentation

A 29-year-old Caucasian male was referred to our upper limb department after reporting persistent left wrist pain for a period of six months without a history of trauma. Over the past eight weeks, the pain has become progressively worse, limiting his daily activities. From his past medical history, the patient was free of disease. The pain was most prominent at night, disturbing the patient’s sleep, and was occasionally relieved with the use of analgesics and oral anti-inflammatory drugs. Splinting has also been tried to alleviate pain without long-lasting favorable results.

On physical examination, local tenderness over the volar area at the carpometacarpal joint of the thumb was evident with mild swelling. The range of movement of the carpometacarpal joint was slightly decreased compared to the contralateral side.

**Figure 1:**
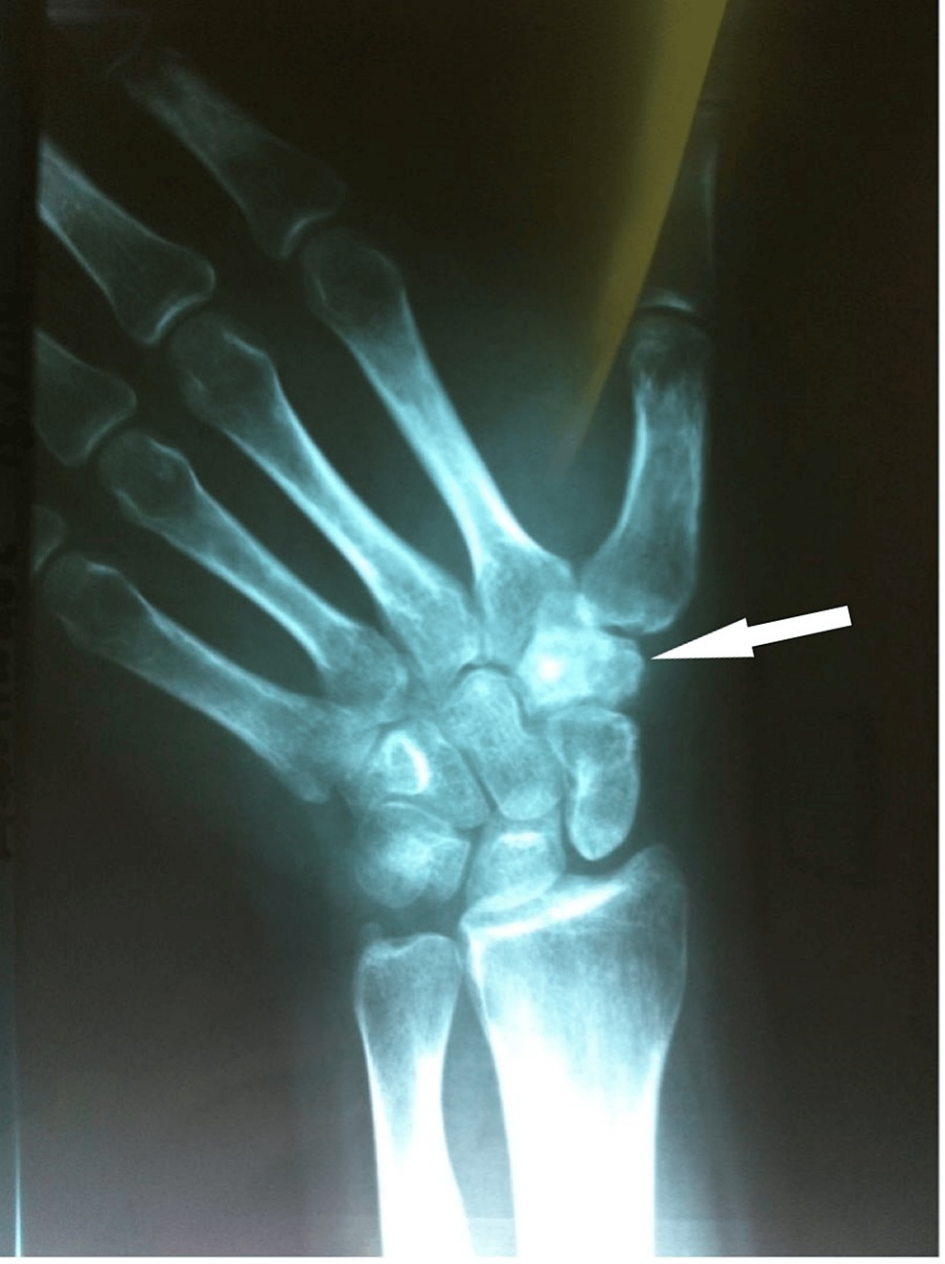
Initial radiograph Anteroposterior radiograph of the left wrist showing bone sclerosis of the trapezium.

**Figure 2:**
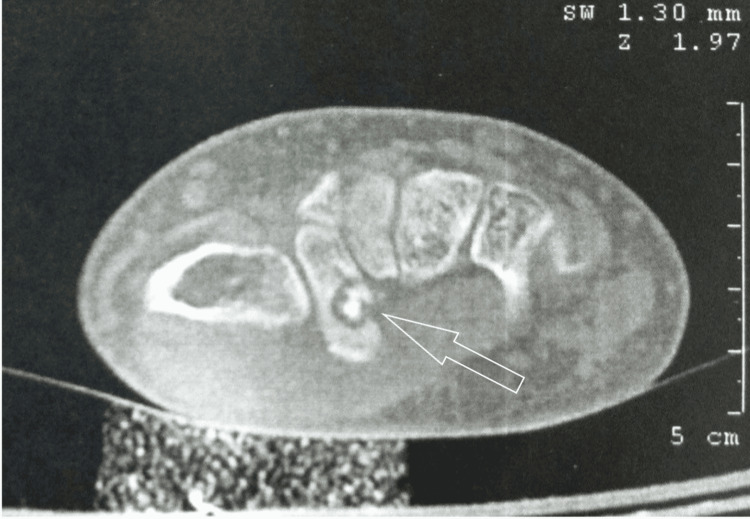
CT tomography Computed tomography reveals the characteristic dense nidus, surrounded by a radiolucent halo.

**Figure 3:**
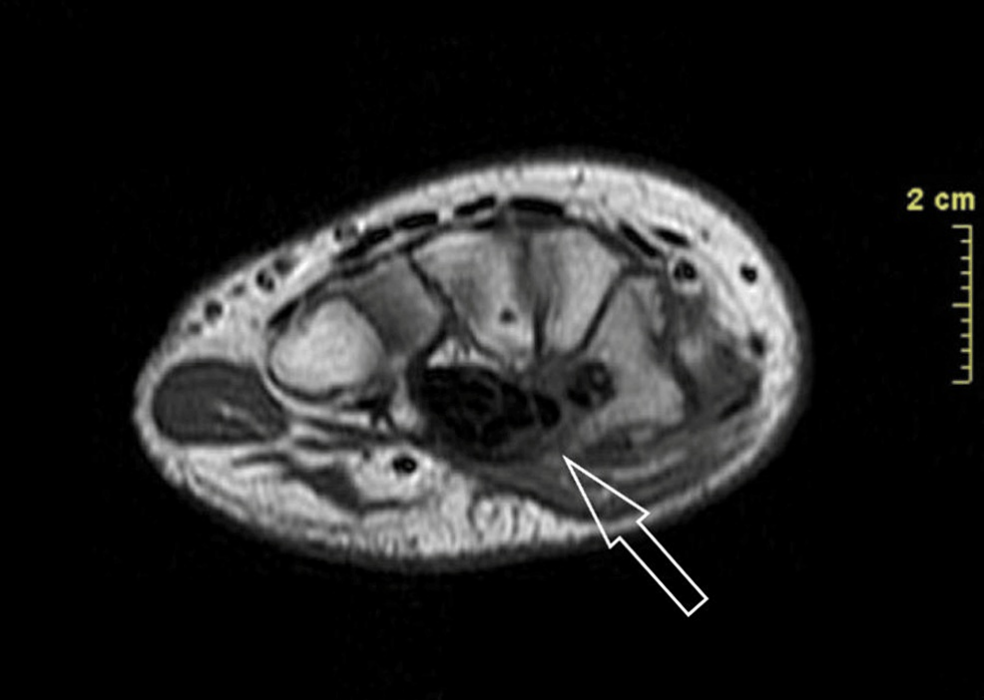
Wrist MRI MRI of the affected wrist. A hypointense lesion of the trapezium is visible.

A plain radiograph was obtained initially, revealing bone sclerosis (Figure 1). Supportive imaging included CT and MRI. On CT, the characteristic low attenuation nidus with surrounding sclerosis was evident (Figure 2). Magnetic resonance imaging (MRI) revealed edema and a hypointense lesion of the trapezium (Figure 3).

**Figure 4:**
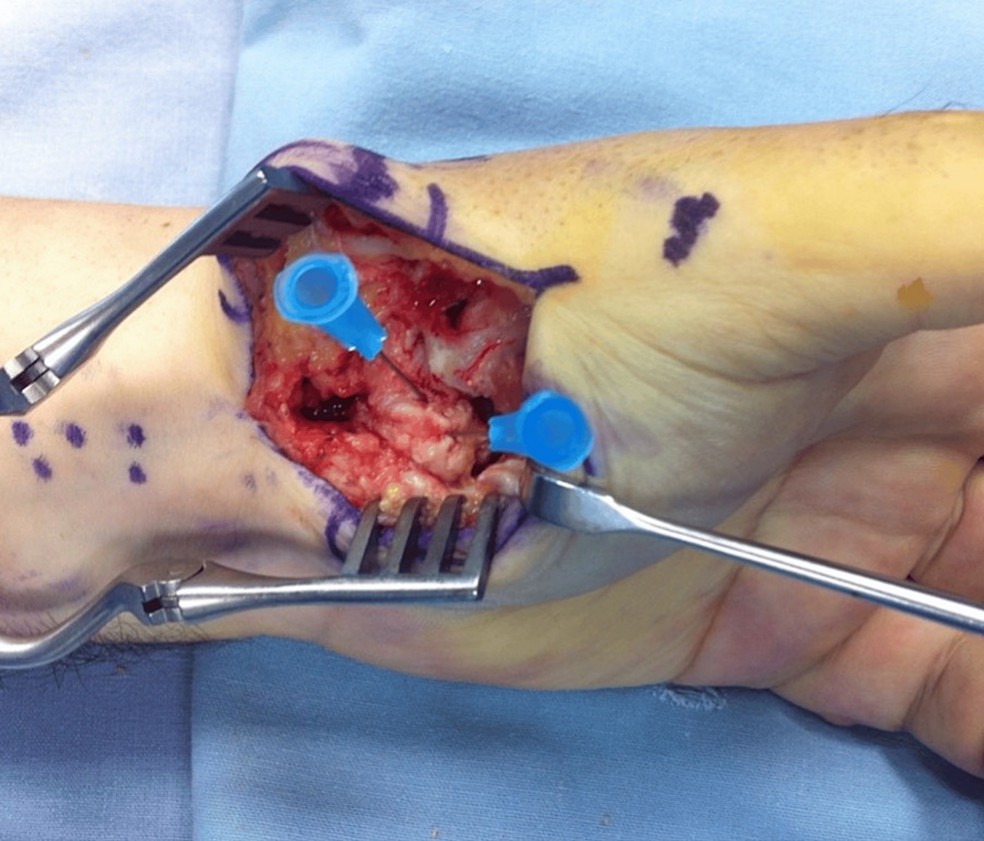
Intra-operative photographs A volar incision provided great exposure to the affected bone.

**Figure 5:**
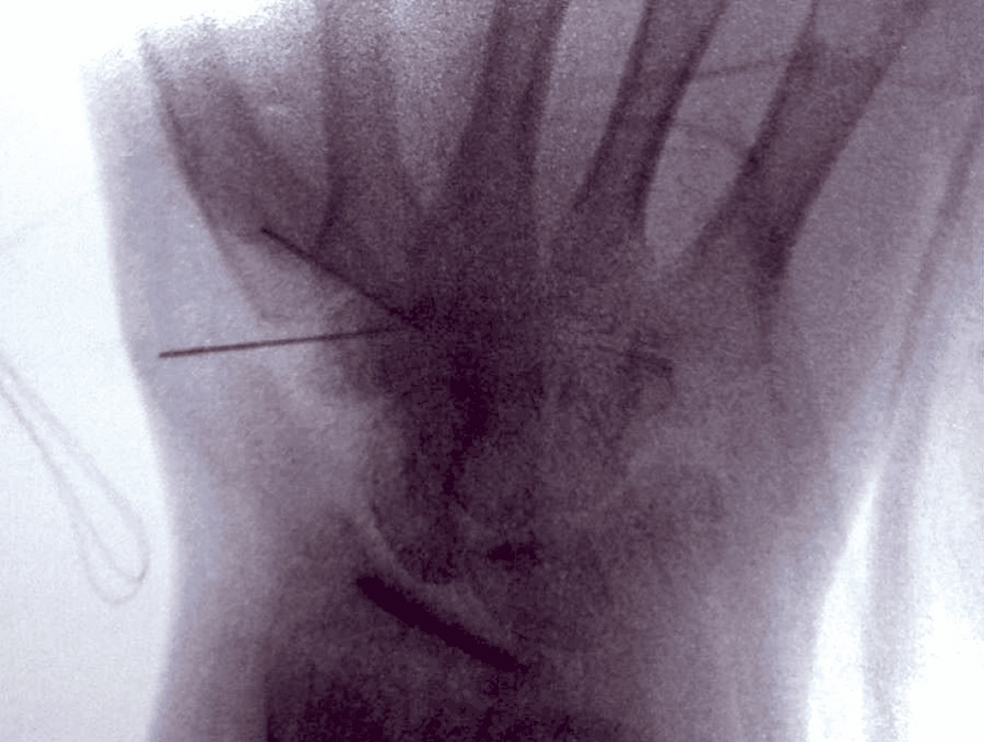
Intra-operative C-arm radiograph The nidus was tagged with two needles and marked with the aid of C-arm fluoroscopy.

**Figure 6:**
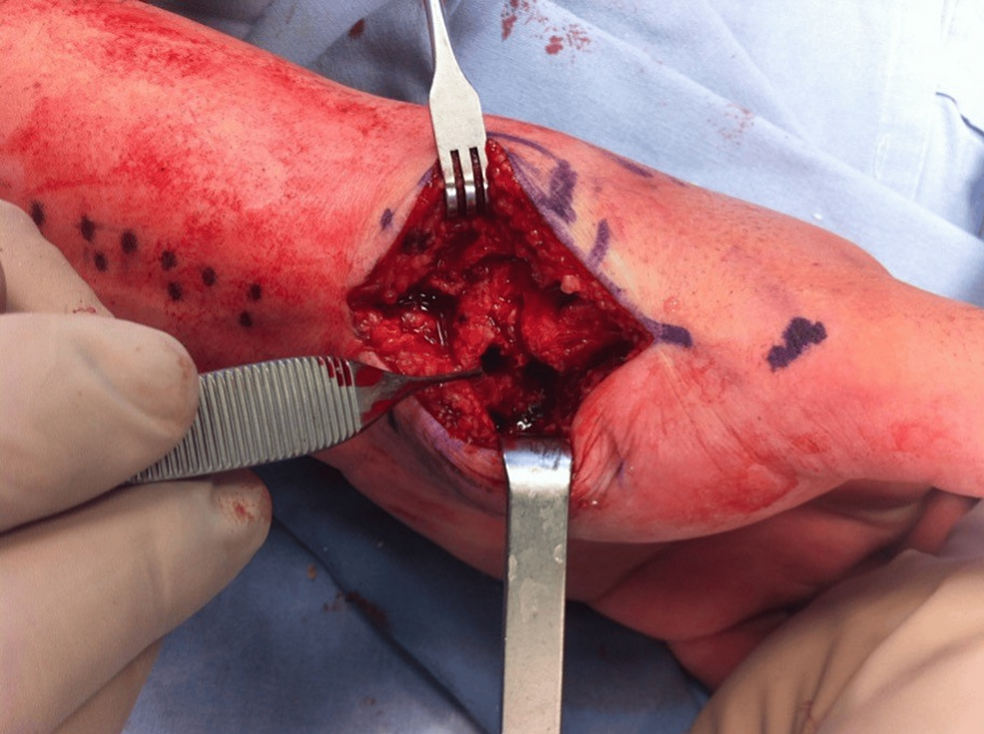
Intra-operative photograph Enbloc excision was performed without any complications.

Based on the above clinical features and imaging findings, an excisional biopsy through a volar incision was decided (Figure 4). The nidus was marked with C-arm fluoroscopy (Figure 5), and enbloc excision was performed (Figure 6). The removed bone was sent for histologic examination, which confirmed our diagnosis.

**Figure 7:**
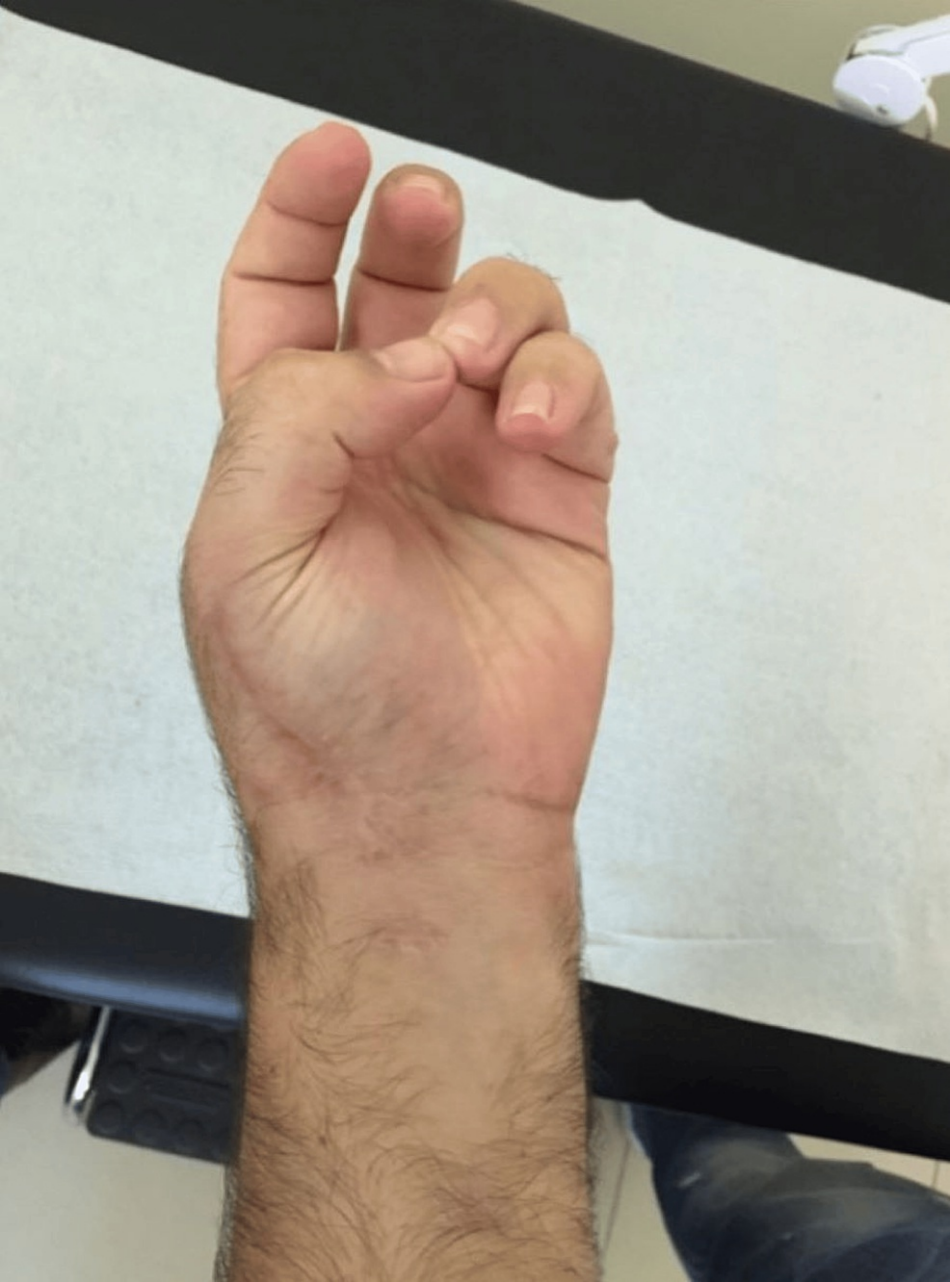
Post-operative pictures Post-operative pictures showing full range of motion of the affected hand’s fingers at the six-month follow-up.

**Figure 8:**
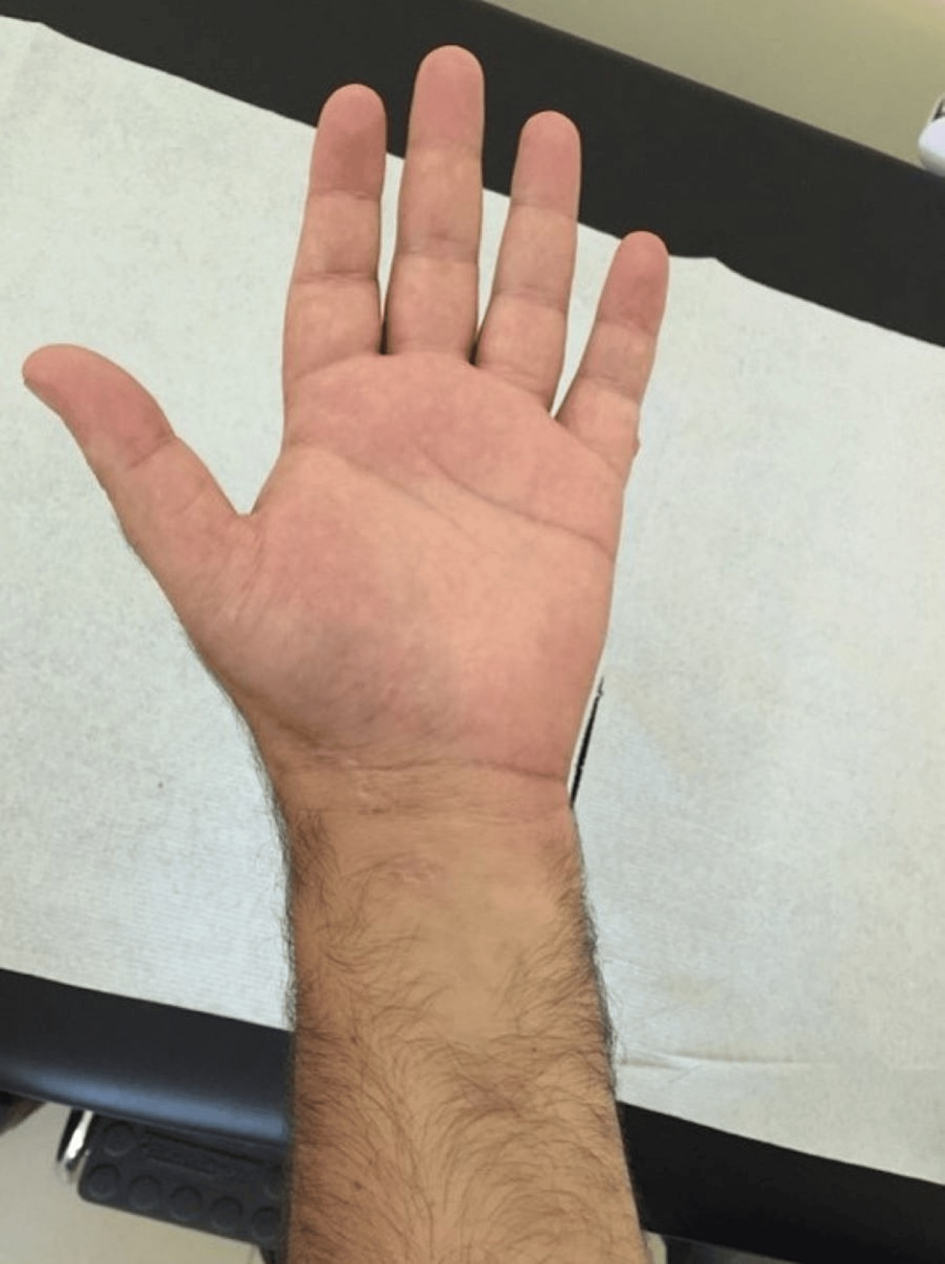
Post-operative pictures Post-operative pictures showing full range of motion of the affected hand’s fingers at the six-month follow-up.

**Figure 9:**
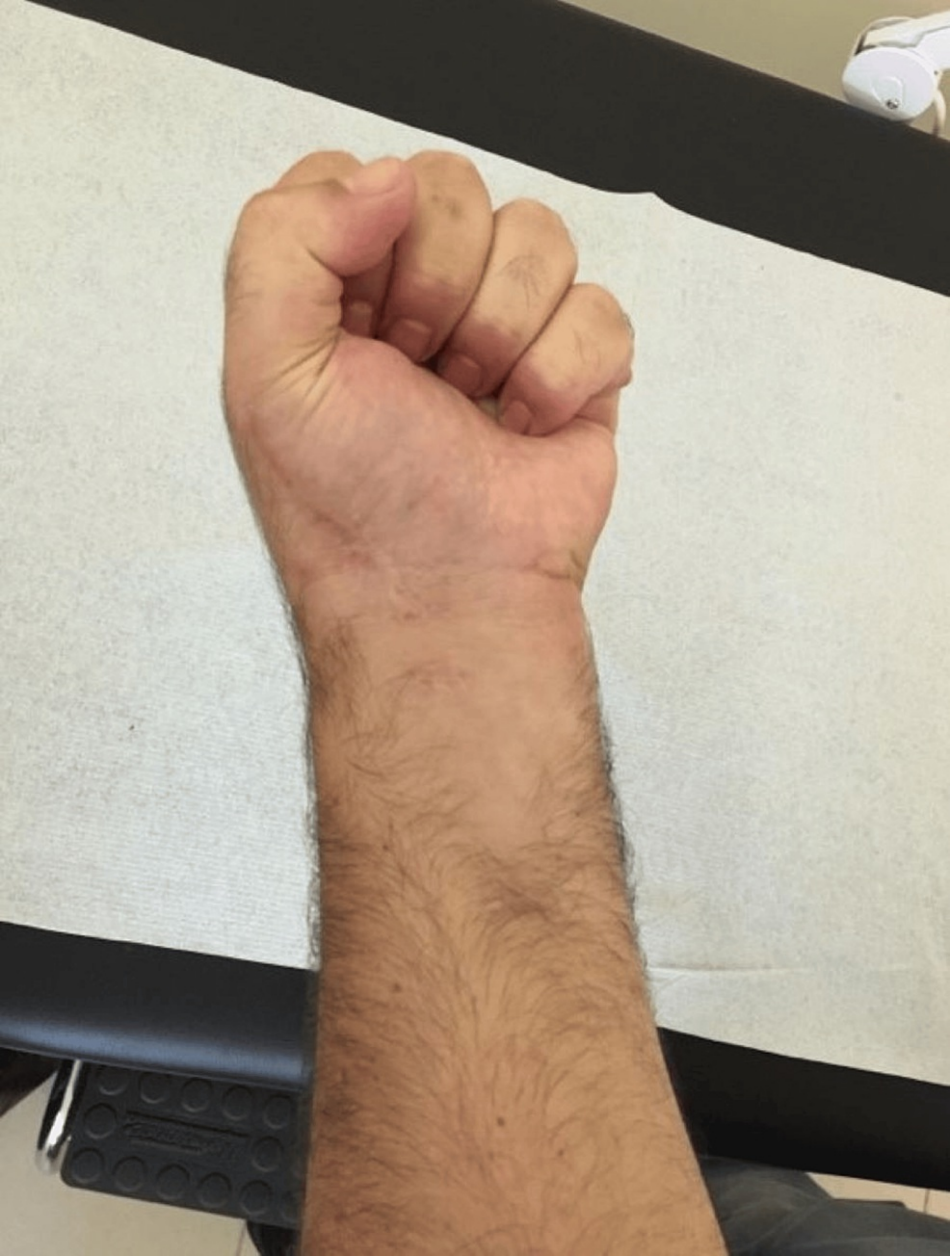
Post-operative pictures Post-operative pictures showing full range of motion of the affected hand’s fingers at the six-month follow-up.

**Figure 10:**
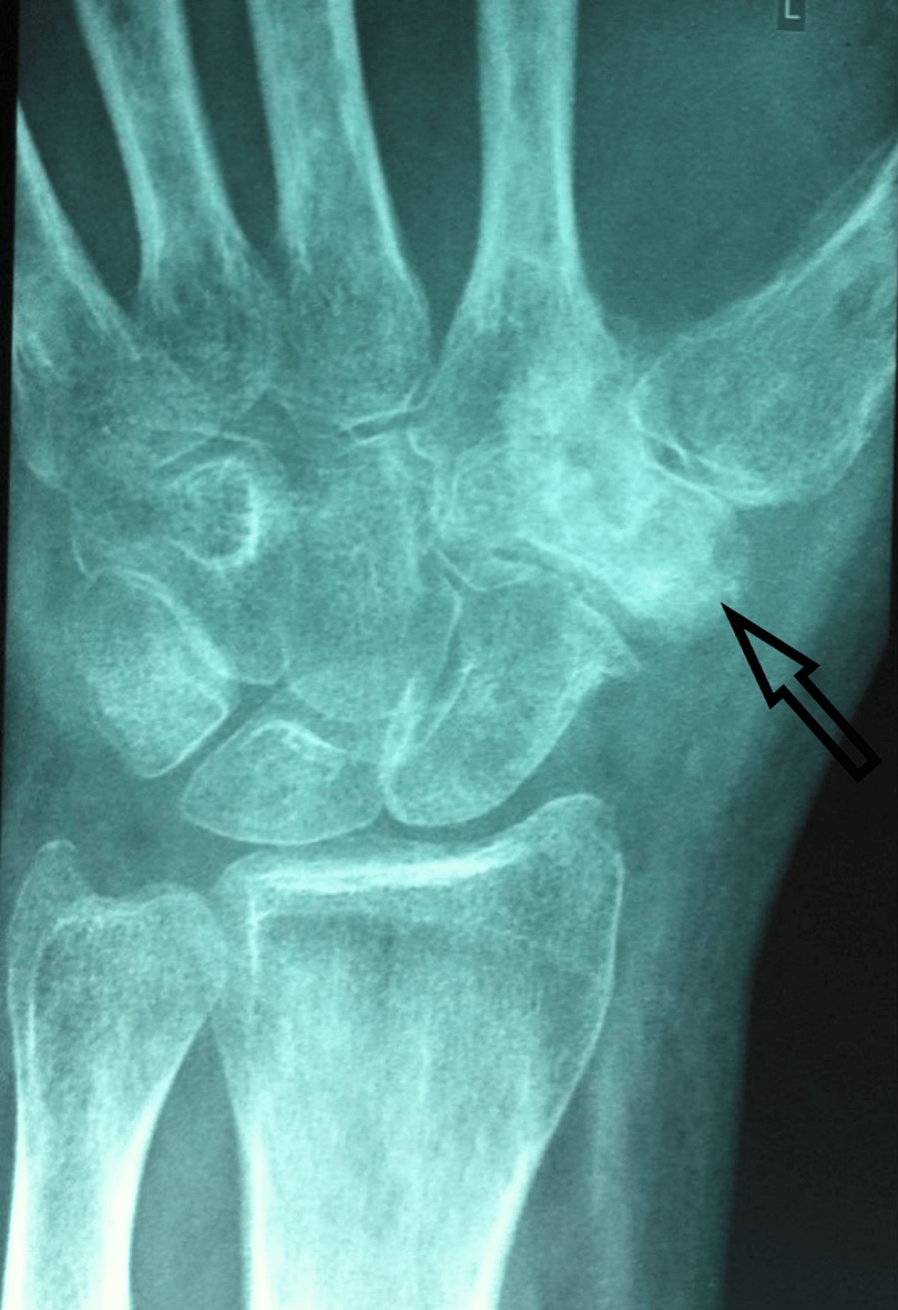
Post-operative anteroposterior radiograph A post-operative anteroposterior radiograph of the left wrist at 12 months showed mild arthritic changes but no recurrence of the lesion.

The patient showed immediate improvement in his symptoms, and six months postoperatively, he has returned to his previous level of activity without restrictions and with a full range of motion (Figures 7-9). Plain radiographs revealed complete excision without recurrence at 12 months (Figure 10).

## Discussion

Osteoid osteoma is a common benign lesion, representing 10% to 12% of all benign bone tumors and 3% of all primary bone tumors [2]. Males in their second decade of life are mostly affected, and in more than 50% of the cases, the metaphysis and diaphysis of long bones are involved [3,7,8]. The first case of osteoid osteoma of the trapezium was reported in 1976 in a 16-year-old patient [9]. Other common sites include the spine, upper extremity, hands, feet, and pelvis [3]. Although the carpus is a rare location for an osteoid osteoma to occur, Murray et al. report 11 osteoid osteomas in 44 primary carpal tumors detected, reviewing 26,800 bone neoplasms in total [7]. None of the 44 carpal tumors were located at the trapezium [7].

The most frequent clinical feature of osteoid osteoma is local pain, which is worsened at night and is typically relieved with non-steroidal anti-inflammatory drugs [10]. Local tenderness and swelling may also be present, as in our case. Other clinical manifestations of osteoid osteoma, depending on its location, are the presence of bone deformity, gait disturbance, limb length discrepancy, and synovitis [8]. When present in the carpal bones, the differential diagnosis includes carpal tunnel syndrome, tenosynovitis, arthritis, infection, reflex sympathetic dystrophy, and neuroma [7].

Standard diagnostic imaging is routinely obtained and consists of plain radiographs, CT, MRI, and bone scintigraphy. The typical radiolucent nidus can be obvious on plain radiographs in the case of a cortical lesion but may be absent in medullary lesions, especially in locations such as the carpus where radiography alone may not be sufficient to assess the bone. In this case, a sclerotic bone was found on the initial radiographic evaluation, prompting further investigation. MRI is reportedly less useful during the diagnostic procedure as it may lead to misdiagnosis and fail to identify small nidi [4,11]. However, new techniques being applied, such as increased spatial resolution and contrast enhancement, offered this imaging choice the advantage of visualizing the nidus and even gaining a lead versus CT when assessing the bone marrow and soft tissue edema and synovitis [3,4]. Bone scintigraphy may be of value due to the increased radionuclide uptake of the nidus and the less intense reactive bone, forming the 'double density sign,' which is diagnostic for osteoid osteoma [3]. CT is the method of choice for a correct diagnosis, especially in the intraarticular location of the nidus or in cases where radiography alone cannot reveal an obvious typical lesion. The typical finding on CT, as illustrated in our patient as well, is the presence of a low-attenuated nidus with surrounding sclerosis [8]. A highly sensitive and specific finding is the presence of fine, low-density, linear, vascular channels surrounding the osteoid osteoma [3].

To the best of our knowledge, only a few cases of osteoid osteomas located in the trapezium bone have been published in recent literature. Hundley [9] reported in 1976 the first case of osteoid osteoma of the trapezium in a 16-year-old boy, treated with surgical excision. Complete pain relief with no recurrence was documented. Since then, only six additional cases have been reported. Helbing and Blauth [12] in 1985 and Cichý [13] in 1997 reported the next two cases; however, complete data could not be recovered. In 2010, Bostan et al. [5] reported an osteoid osteoma in a 25-year-old patient with a 12-month history of wrist pain that was worse at night and had not responded to treatment with non-steroidal anti-inflammatory drugs (NSAIDs) and orthoses. Over the dorsoradial portion of the hand, edema was identified during a clinical examination. A sclerotic nidus surrounded by radiolucent osteoid tissue was seen in the CT scan, and an accompanying bone marrow edema with a localized lesion of the trapezium was visible on an MRI scan. An excisional biopsy was used to treat the patient. Following surgery, the patient's pain immediately subsided, and no recurrence was found during follow-up. In 2017, Park et al. [4] described an osteoid osteoma in a 29-year-old patient who had been initially treated for calcification periarthritis with several steroid injections. An ulnar deviated X-ray revealed a sclerotic bone lesion, suspicious for osteoid osteoma. The patient was treated with curettage, experiencing immediate improvement in pain and no recurrence at 12-month follow-up. A 34-year-old woman with osteoid osteoma was initially misdiagnosed with carpometacarpal arthritis, according to a report by Roberts et al. [6] in 2017. In this instance, an MRI scan revealed a hypointense circular lesion along the dorsal portion of the trapezium. To further characterize the lesion, a CT scan was performed. Following the tumor excision, there was complete pain relief and no indication of a recurrence. In 2022, Gravina et al. [3] reported a case of a 19-year-old male with intense, dull, and persistent pain at the right thumb basal joint for one year. Initial X-rays were normal, while an MRI revealed an intense signal corresponding to the trapezium and a diffuse edema of the surrounding tissue of the trapezium. An additional CT scan showed a sclerotic nidus surrounded by a cortical reaction, confirming the diagnosis of an osteoid osteoma. Enucleation of the nidus using curettes, followed by bone grafting using bioactive glass to fill the bone loss, showed notable improvement in Pinch test scores, Kapanji scores, and the Visual Analogue Scale (VAS) scale after a follow-up period of 12 months.

While in other anatomic locations, different treatment options can be discussed for the management of such lesions, surgical or conservative, the treatment of osteoid osteoma of the carpal bones is surgical excision of the nidus [5-7]. Another option indicated for large lesions involving the trapeziometacarpal joint is trapeziectomy. Recurrence is extremely rare and is mainly attributed to incomplete excision during curettage [6].

**Table 1:** Literature review of osteoid osteomas of the trapezium

Authors	Demographics, clinical presentation	Diagnostic tools	Treatment method	Outcomes
Cichý et al. [13]	N/A	Bone scintigraphy, MRI, CT	N/A	N/A
Hundley et al. [9]	Male, 16y. N/A	Radiographs: progression of the lesion from normal-appearing bone to bone with typical changes of osteoid osteoma.	Surgical excision	Complete and instant pain relief
Gravina et al. [3]	Male, 19y. Intense, dull and persistent pain at the right thumb basal joint for 1 year.	Radiographs were normal. MRI: intense signal corresponding to the trapezium and a diffuse edema of the surrounding tissue CT: sclerotic nidus surrounded by a cortical reaction.	Enucleation of the nidus using curettes though an ‘S’ incision on the radial volar side. Bone defect was filled with bioglass.	Notable improvement in Pinch test, Kapanji scores and VAS scale after 12-month follow-up.
Roberts et al. [6]	Female, 34y. Two-year history of pain at the base of the right thumb and radial side of the wrist. No history of trauma. Pain sometimes woke her up.	Initial radiographs were read as unremarkable. MRI: circular hypointense lesion along the dorsal aspect of the trapezium suspicious for an osteoid osteoma. CT: same defect as MRI, yet slightly larger.	Surgical resection through a vertical dorsal incision. No additional bone grafting.	Complete pain relief. No recurrence to date.
Park et al. [4]	Male, 29 y, severe thumb basal joint pain.	Ulnar deviated radiograph	Curettage	Immediate improvement in pain and no signs of recurrence after a 12-month follow-up.
Helbing et al. [12]	N/A	N/A	N/A	N/A

## Conclusions

In conclusion, osteoid osteoma of the trapezium is an almost unknown entity for the unaware physician. A thorough history may reveal the characteristics of the pain, such as chronicity and nocturnal appearance, which should raise awareness. The diagnosis is generally reached by plain radiographs and CT tomography. The definitive diagnosis is set by histological examination. Treatment consists of cutterage of the osteoid osteoma and trapeziectomy in larger tumors. In that case, bone grafting and bone substitutes may be necessary. We present the eighth report of this lesion in the literature with the aim of raising suspicion about the presence of this entity in patients with long-lasting, disabling, undiagnosed wrist pain.
